# Training response inhibition to food is associated with weight loss and reduced energy intake

**DOI:** 10.1016/j.appet.2015.06.009

**Published:** 2015-12-01

**Authors:** Natalia S. Lawrence, Jamie O'Sullivan, David Parslow, Mahmood Javaid, Rachel C. Adams, Christopher D. Chambers, Katarina Kos, Frederick Verbruggen

**Affiliations:** a School of Psychology, College of Life and Environmental Sciences, University of Exeter, Exeter, EX4 4QG, UK; b School of Psychology, Cardiff University, Park Place, Cardiff, CF10 3AT, UK; c University of Exeter Medical School, Barrack Road, Exeter, EX2 5DW, UK

**Keywords:** Response inhibition, Cognitive training, Weight loss, Energy intake, Food liking, Disinhibition

## Abstract

The majority of adults in the UK and US are overweight or obese due to multiple factors including excess energy intake. Training people to inhibit simple motor responses (key presses) to high-energy density food pictures reduces intake in laboratory studies. We examined whether online response inhibition training reduced real-world food consumption and weight in a community sample of adults who were predominantly overweight or obese (N = 83). Participants were allocated in a randomised, double-blind design to receive four 10-min sessions of either active or control go/no-go training in which either high-energy density snack foods (active) or non-food stimuli (control) were associated with no-go signals. Participants' weight, energy intake (calculated from 24-h food diaries), daily snacking frequency and subjective food evaluations were measured for one week pre- and post-intervention. Participants also provided self-reported weight and monthly snacking frequency at pre-intervention screening, and one month and six months after completing the study. Participants in the active relative to control condition showed significant weight loss, reductions in daily energy intake and a reduction in rated liking of high-energy density (no-go) foods from the pre-to post-intervention week. There were no changes in self-reported daily snacking frequency. At longer-term follow-up, the active group showed significant reductions in self-reported weight at six months, whilst both groups reported significantly less snacking at one- and six-months. Excellent rates of adherence (97%) and positive feedback about the training suggest that this intervention is acceptable and has the potential to improve public health by reducing energy intake and overweight.

## Highlights

• We examine whether response inhibition training alters real-world eating behaviour.
• Food no-go training (active) reduces energy intake, weight and food liking.
• Non-food no-go training (control) has no effect pre-to post-intervention.
• Both groups show reduced snacking at one and six-month follow-ups.
• Active training is associated with self-reported weight loss at six-months.

## Graphical abstract

**Figure:**
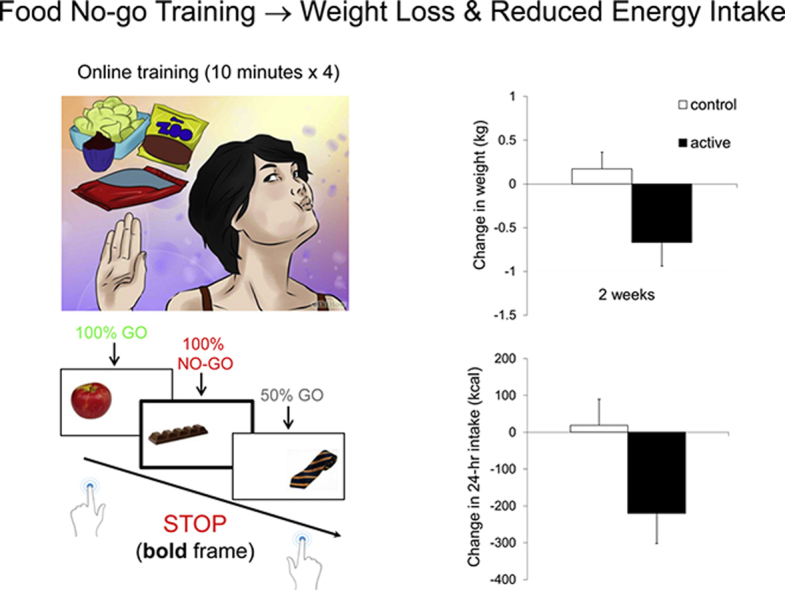

## 1 Introduction

The prevalence of overweight and obesity has shown an increase over the past 30 years and the majority of adults in the US and UK are now overweight or obese (65–70%; Flegal, 2005; Wang & Beydoun, 2007). Overeating in a food-rich environment is a key contributor to rising obesity levels (Hill, Wyatt, Reed, & Peters, 2003) begging the question, how can we support people to reduce their over-consumption of food? Weight management interventions need to include behaviour change strategies that improve eating behaviour and reduce energy intake (Cavill & Ells, 2010).

Several models of self-control, notably dual process models, indicate that one important determinant of behaviour toward palatable, high-energy density foods is the unintentional elicitation of motor impulses towards these foods (Hofmann, Friese, & Wiers, 2008; Metcalfe & Mischel, 1999; Strack & Deutsch, 2004). These fast-acting, associatively-mediated impulses are thought to be regulated by a slow, controlled, reflective system that enables explicit goals and personal standards to influence behaviour, e.g. via top-down cognitive control. The strength of the impulses towards food and whether or not they give rise to consumption, depends on the interaction between the impulsive and reflective system, which are reflected in individual differences in food reward-sensitivity and self-control, respectively (Hofmann, Friese, & Roefs, 2009; Lawrence, Hinton, Parkinson, & Lawrence, 2012). Individuals who show a strong reward-related response to foods combined with low levels of self-control are particularly susceptible to overeating and overweight, whereas those with effective self-control appear to be protected (Lawrence et al., 2012; Nederkoorn, Houben, Hofmann, Roefs, & Jansen, 2010). This supports substantial evidence linking behavioural measures of poor self-control, namely motor response inhibition measured using stop-signal and go/no-go tasks (Verbruggen & Logan, 2008a), to overeating and overweight (Batterink, Yokum, & Stice, 2010; Guerrieri et al., 2007; Houben, Nederkoorn, & Jansen, 2014; Nederkoorn, Braet, Van Eijs, Tanghe, & Jansen, 2006a, 2012; 2006b). Thus regulation or reduction of food-related impulses seems to be required to control eating behaviour in our plentiful food environment and is a promising target for weight management interventions aimed at the habitual or impulsive system (Marteau, Hollands, & Fletcher, 2012; Van't Riet, Sijtsema, Dagevos, & Bruijn, 2011).

Laboratory studies suggest that the inhibition of responses to specific stimuli can be trained using consistent stimulus-stop or no-go associations, resulting in automatic response inhibition (Verbruggen & Logan, 2008b). More specifically, response inhibition is said to be 'automatic' when it is triggered by the retrieval of stimulus-stop associations from memory (Logan, 1988; Verbruggen & Logan, 2008b). Training response inhibition to specific snack food stimuli reduces the subsequent intake, choice and self-served portion size of those foods (Houben, 2011; Houben & Jansen, 2011, 2015; Lawrence, Verbruggen, Morrison, Adams, & Chambers, 2015; Van Koningsbruggen, Veling, Stroebe, & Aarts, 2014; Veling, Aarts, & Papies, 2011; Veling, Aarts, & Stroebe, 2013a; 2013b). These training effects are particularly pronounced in restrained eaters (Houben & Jansen, 2011; Lawrence et al., 2015; Veling et al., 2011), who are prone to overeating when disinhibited and frequently attempt to diet with or without success (Lowe, 1993). Response inhibition training effects on food choices are also stronger in those with a high appetite and in those who frequently consume the palatable, high-energy density ‘no-go’ training foods (Veling et al., 2013a; 2013b), suggesting stronger training effects in those most vulnerable to overeating and overweight.

In terms of the potential mechanisms underlying the effects of food response inhibition training on reduced food intake and choice, findings suggest that stimuli associated with response inhibition show reductions in motor excitability and reward value (Verbruggen, McLaren, & Chambers, 2014). For example, the automatic motor impulses activated by stimuli, and in particular palatable food cues, are reduced following response-inhibition training (Chiu, Aron, & Verbruggen, 2012; Chiu, Cools, & Aron, 2014; Houben & Jansen, 2015; Veling et al., 2011; Verbruggen & Logan, 2008a), and this may be associated with reduced food consumption (Houben & Jansen, 2015; Veling et al., 2011; *cf* Houben, Havermans, Nederkoorn, & Jansen, 2012). In terms of reward value, affective cues associated with no-go responses show a reduction in rated valence (Doallo et al., 2012; Veling, Holland, & van Knippenberg, 2008; Veling et al., 2013a) and more negative implicit affective reactions (Houben et al., 2011; 2012; Veling & Aarts, 2009). If food-associated response inhibition training effectively boosts automatic motor inhibition and reduces the reward value associated with food cues, it could help at-risk individuals control their food intake. This study therefore examined the effects of repeated sessions of food-associated no-go training, delivered via the internet, on a range of ‘real world’ measures of eating behaviour.

### 1.1 The present study

Previous studies have employed both stop-signal and go/no-go tasks to train associations between foods and motor inhibition. Stop-signal tasks impose a delay between the stimulus and a stop signal and so require the cancellation of an initiated response, whereas the no-go signal is presented at the same time as the stimulus so a response should not be initiated (Schachar et al., 2007). Recent findings from our lab suggest that food no-go training (‘action restraint’) may be more effective than stop-training in reducing food intake (Adams, Verbruggen, Lawrence, & Chambers, 2014, discussed in Lawrence et al., 2015) so here we used a food go/no-go task based on our lab studies, in which high-energy density foods (greater than 4 kcal/g) were consistently associated with no-go signals and healthy, lower-energy density foods were consistently associated with go-signals.

Lab studies to date have compared food response inhibition training to control conditions requiring either consistent or inconsistent ‘go’ responses to foods, which may have inadvertently increased approach towards, and intake of food in control participants (Lawrence et al., 2015; Schonberg et al., 2014). To avoid this potential confound, the present study employed a control condition in which participants were trained to inhibit responses to non-food pictures and were never exposed to pictures of high-energy density foods. As the active group were repeatedly exposed to images of high-energy density food (paired with no-go responses) during training, mere exposure effects would predict *increased* food intake in the active relative to control group (Fedoroff, Polivy, & Herman, 1997); any reduction is therefore likely due to the food-associated inhibition training, which may include related processes such as food cue exposure with response prevention.

Participants completed four sessions of food-related (versus control) no-go training in one week and effects on weight loss, energy intake and daily snacking frequency were measured. We also examined training effects on two variables used in previous laboratory studies - subjective ratings of food images (Veling et al., 2013a) and food intake in a taste test presented immediately following an additional training session (Lawrence et al., 2015). Finally, we measured the longer-term effects of training by contacting participants one month and six months after their final session and asking them to provide their current weight and snacking frequency. We predicted that the active group would show a greater reduction than the control group in weight, snacking frequency, energy intake and snack food intake in the taste test. We also expected a larger reduction (devaluation) in subjective ratings of the high-energy density (no-go) foods in the active relative to control group.

Since the current research was conducted a similar study has been published that associated stop signals with many palatable foods and drinks in a dieting sample to facilitate weight loss (Veling, Koningsbruggen, Aarts, & Stroebe, 2014). Four weekly training sessions delivered via the internet resulted in weight loss in the active group relative to a control group that, like here, was trained to inhibit to non-food images. The Veling et al. (2014) sample included predominantly young, healthy females, and the effects of no-go training on weight loss were greater in higher BMI participants. Veling et al. (2014) suggested that further work was required to determine whether the food no-go intervention is associated with weight-loss over the longer term, whether it is effective in more overweight participants, and what the possible mechanisms of training effects are before this promising intervention can be recommended as a weight-loss tool. All of these factors were addressed in the current study, which recruited predominantly middle-aged overweight or obese adults, followed them up over a longer period of time and examined some putative mechanisms underlying training effects. The current study is therefore relevant in assessing the potential of food no-go training on weight loss and eating behaviour in those most in need of intervention.

## 2 Material and methods

### 2.1 Design

Participants were randomly allocated to one of two groups, with a between group factor of response inhibition training (active versus control) and a within subjects factor of time (pre versus post-intervention). Unless otherwise specified, mixed-effects ANOVAs were conducted on dependent variables using SPSS 21 (IBM Corp, 2012). All data files are deposited in the University of Exeter's Open Research Exeter repository under the following identifier: (http://hdl.handle.net/10871/17621)

### 2.2 Participants

Participants were recruited from two community samples of adults following completion of an online screening questionnaire to assess eligibility (see supplementary methods). Briefly, eligibility required that participants were aged 18–65, had a Body Mass Index (BMI) based on self-reported height and weight of at least 18.5 (healthy range and above), consumed some of the ‘no-go’ snack foods (see below) at least three times per week, and reported some disinhibition (loss of control) over eating (Three Factor Eating Questionnaire subscale, Stunkard & Messick, 1985). Our aim was to examine training effects in individuals with vulnerability factors for overeating and overweight, so we included participants who were already overweight *and* those of a healthy weight who reported some unhealthy snacking habits and loss of control over their food intake (who may be at risk for future weight gain or other negative health consequences of consuming unhealthy snacks; see supplementary methods for further information). Study exclusion criteria included allergies to the foods given during the taste test (chocolate and crisps), and factors that could affect weight but were unrelated to the intervention, namely smoking/recent smoking cessation, enrolment in a formal weight-loss programme, use of weight-loss medication, metabolic disorders or other health conditions affecting weight.

**Fig. 1:**
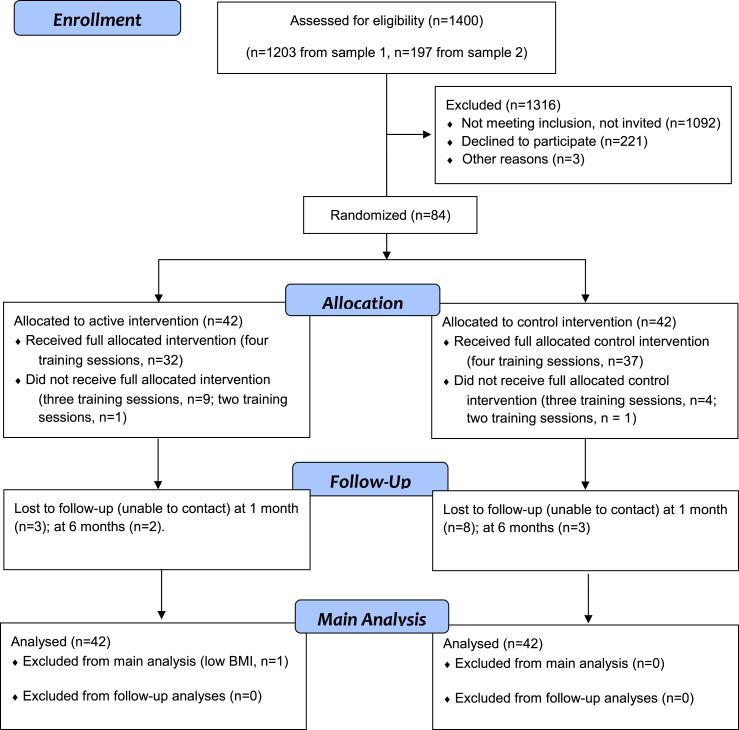
Recruitment flow diagram showing numbers of participants included in each intervention group at each stage of the study (see supplementary methods for details of each sample).

A total of 1400 participants completed the online survey and 308 met eligibility criteria and were invited to participate (see recruitment flow chart, Fig. 1). Suitable participants with a BMI greater than 25 (overweight or obese) and disinhibition scores above the sample median (5) were invited to participate first, followed by those with lower (healthy) BMIs and lower disinhibition scores. Of the 308 invited participants, 87 (64 female) were recruited into the study and 84 were randomised to receive the active or control intervention (Fig. 1). Three participants dropped out (attrition rate 3.4%) for reasons of poor health or time commitments prior to being randomized; they were excluded as we had no data from them beyond the baseline week. One participant in the active condition was excluded due to a low measured BMI at baseline (below 18.5), which was not detected at screening. All of the remaining 83 participants completed at least two training sessions during the intervention week (one with the researcher, one on their own) and 82% completed all four training sessions (Fig. 1). All 83 participants were retained in the main analysis, consistent with an intention-to-treat analysis (Hollis & Campbell, 1999). Ethical approval for the trial was granted by the Psychology Department Board of Ethics at the University of Exeter and all participants gave written informed consent to participate.

### 2.3 Measures

*Weight*. Participants' weight in kilograms was measured at screening, baseline, post-intervention (two weeks after baseline reading) and at one-month (six weeks after baseline reading) and six-month follow-up. Weight was measured by a researcher at baseline and post-intervention using a set of Salter digital bathroom scales, and by the participant at screening and follow-up.

### 2.4 Snacking frequency

Participants completed a Food Frequency Questionnaire (Churchill & Jessop, 2011), rating how often eight common snack foods were consumed over the previous month using an eight-point scale (ranging from 8 = “4 or more times a day”, to 2 = “1 to 3 times a month” and 1 = “less often or never”). Scoring was reversed from Churchill and Jessop (2011) so that a high score indicated more snacking, and a score for the four ‘no-go’ foods (crisps, chocolate, biscuits and cake) at screening was calculated for each participant to determine eligibility. During the intervention phase, participants completed a version of this FFQ that we modified to measure daily frequency of intake on a six-point scale (ranging from 6 = “greater than 4 times today” down to 2 = “once today” and 1 = “not at all”). Participants completed this daily FFQ for one week at both baseline and during the intervention week. Daily scores were again summed over the four no-go foods and a mean daily score for each participant was computed for the baseline and intervention week.

### 2.5 Energy intake

This was calculated from food intake in weight converted to energy intake. Food intake was recorded using multiple hard-copy 24-h food diaries taken from the UK European Prospective Investigation of Cancer (Bingham et al., 1997^1^). Participants recorded all food and drink consumed during two preceding 24-h periods, one mid-week and one at the weekend (Ma et al., 2009), during both the baseline week and the intervention week (four in total). During the intervention week, they were asked to complete their first food diary after completing at least two of their online training sessions and their second food diary after completing all four. The food and drink consumed was converted by a researcher (JOS) to total calories per 24-h diary using an online calorie-counting tool (http://www.mynetdiary.com/).

### 2.6 Food ratings

A computerised stimulus evaluation test programmed in Psychtoolbox (Brainard, 1997) within MATLAB (Mathworks, 2011) measured subjective ratings of all food images included in the active training task on a 100 mm visual analogue scale. Separate blocks examined subjective ratings of image attractiveness and liking of taste (see supplementary methods), consistent with previous work (Veling et al., 2008; 2013a). Participants rated 27 pictures of foods, including 18 from the active training task, i.e. the 9 healthy “go” foods and the 9 high-energy density “no-go” foods. The other 9 images were of novel foods not included in the training task (see supplementary methods) that were included to measure the specificity of any change in ratings of go or no-go foods over time.

### 2.7 Taste test

A taste test was given during the final session to covertly measure consumption of crisps and chocolate immediately after an additional final training session. This test followed the same procedure used in our lab studies (see Lawrence et al., 2015 for a complete description). Briefly, participants were presented with 210 g of chocolate buttons and 100 g of ready salted crisps (these quantities were selected because they appeared as similar portions when presented in two identical large plastic containers) and were asked to taste the products and answer questions about them (taken from Houben, 2011). These included open-ended questions about the sweetness, saltiness and taste of the two foods, along with Likert scales measuring palatability and usual frequency of consumption. This test provided a more immediate and objective measure of training effects on consumption and attempted to replicate previous studies where consumption in the laboratory was measured following a single training session (Houben, 2011; Houben & Jansen, 2011; Lawrence et al., 2015).

### 2.8 Training task

**Fig. 2:**
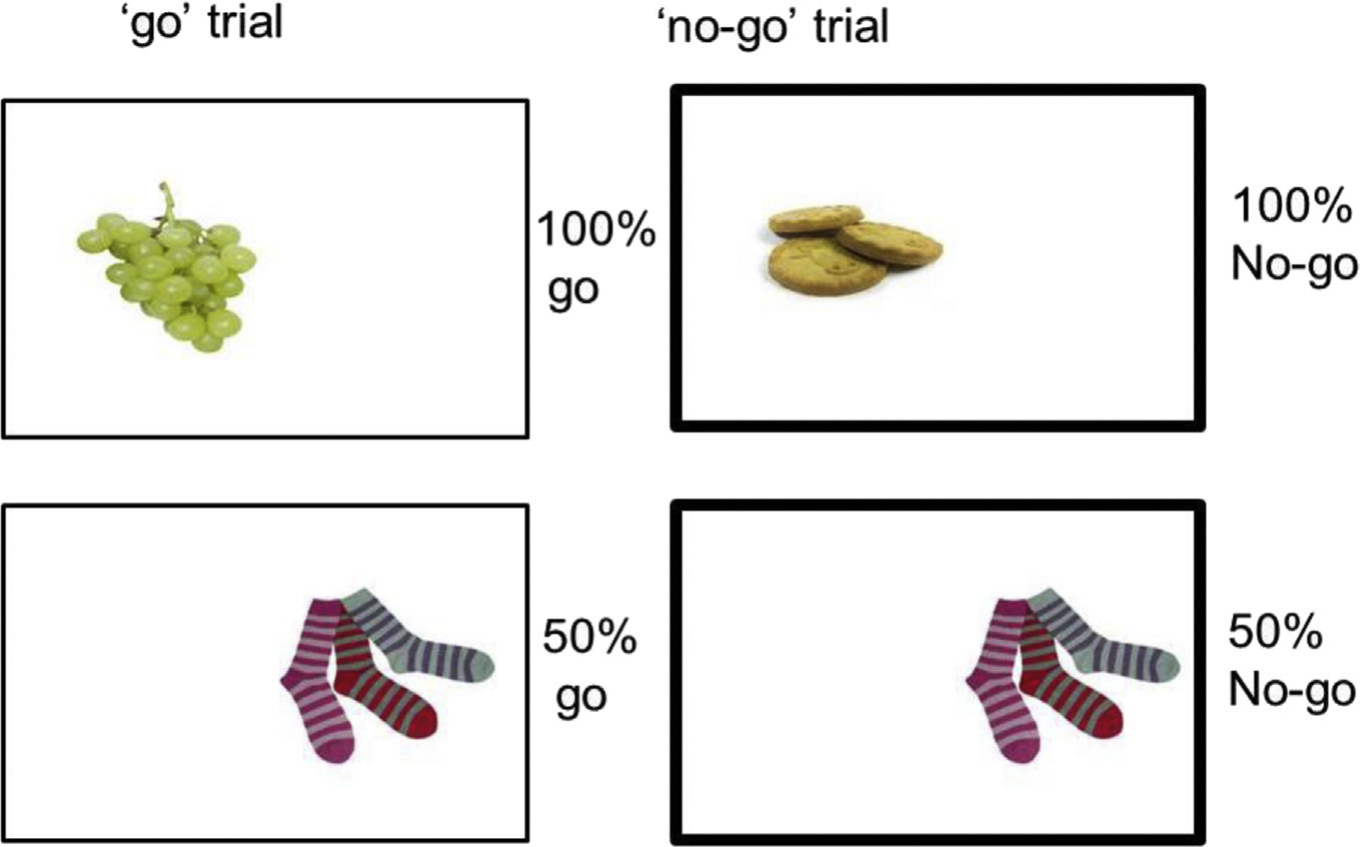
Schematic of the ‘go’ and ‘no-go’ trials for the food associated response inhibition task (active condition). Healthy foods were always presented on go trials, high-energy density foods always on no-go trials (bold frame) and filler images of clothes were associated with no-go signals 50% of the time.

During the online training task, pictures of 18 food (or non-food in the control group) and 18 non-food filler objects were presented individually on the left or right-hand side of a computer screen for 1250 ms followed by a 1250 ms inter-stimulus interval. Participants had to press a button (‘c’ for left and ‘m’ for right) as quickly and accurately as possible to indicate the side of presentation (go-trials; Fig. 2). On half of the trials, the frame surrounding the picture was bold, which was a signal for participants to withhold their response (no-go trials, Fig. 2). Each of the 36 images was presented once per block and participants completed 6 blocks per training session. They were provided with feedback (accuracy and mean go RT) at the end of each block to increase their motivation, and had to press a key to continue with the task.

In the active training task images consisted of 18 foods, of which 9 were healthy (fruit, vegetables, rice cakes) and 9 high-energy density (greater than 4 kcal/g; biscuits, chocolate, crisps – see supplementary methods), along with 18 non-food filler pictures (clothes). In the control training task images consisted of 18 household objects (furniture, stationery, gardening tools) and the same 18 filler clothes pictures. Food and non-food images were matched as closely as possible for size, colour and visual complexity. Each picture was presented inside a rectangular frame against a white background (Fig. 2).

In the active group, high-energy density food images were *always* paired with no-go signals (resulting in 54 high-energy density food-no-go trials per training session), whereas healthy foods were *never* paired with no-go signals (54 healthy food-go trials per training session). The filler images of non-food items (clothes) were equally associated with go and no-go signals (54 go and 54 no-go trials per training session), resulting in 50% no-go trials overall. The inclusion of filler images with unpredictable responses served to make the task more challenging and engaging, and aimed to make the rule less obvious in order to recruit learning in the automatic, associative system, rather than the explicit, rule-based system. In the control group, participants completed an identical task except that pictures of non-food objects replaced the food pictures. The ‘go’ non-food images included electrical items, furniture and buckets and the ‘no-go’ non-food images consisted of DIY tools, gardening tools and stationery. The speed and accuracy of responses to foods and non-foods was measured and stored on a secure server.

### 2.9 Procedure

**Fig. 3:**
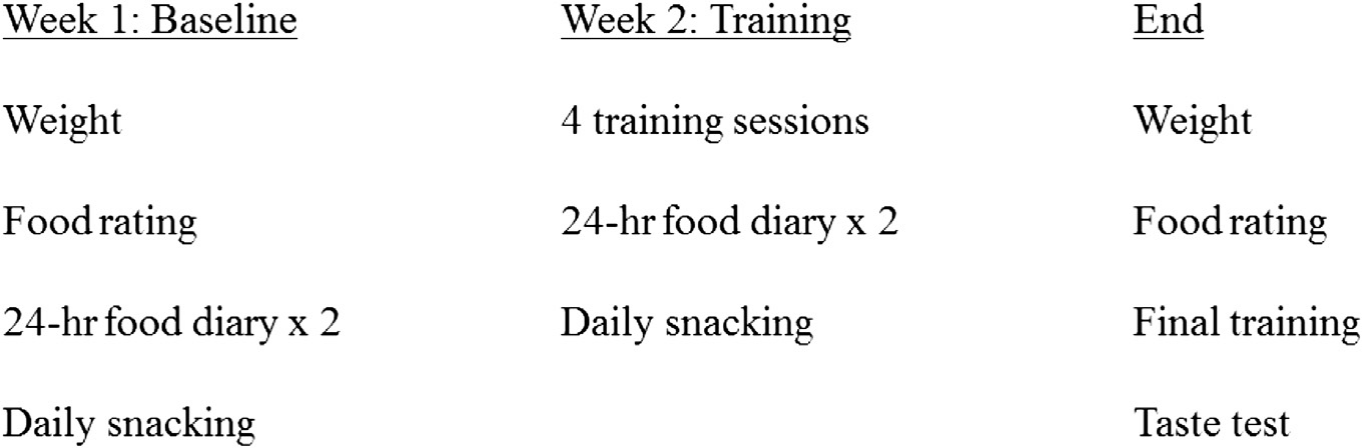
Overview of study procedure during the 2-week pre- and post-intervention phase. Participants were also followed-up remotely one month and six months after the final research session.

A timeline of the study is shown in Fig. 3. Researchers visited participants at their home or place of work to complete an introductory baseline session where participants were informed about how to complete the 24-h diaries and daily FFQs, and gave consent. Participants then performed the baseline stimulus evaluation test on the researcher's laptop, rating food liking and image attractiveness. The researcher then weighed the participant and gave them a set of seven daily FFQs and two 24-h food diaries to complete during the following baseline week (Fig. 3).

After the first week of recording baseline FFQs and 24-h diaries participants started their online response inhibition training at their home or place of work. The researcher showed the participant how to access the online training, read them the instructions and gave them a unique identification code. When participants were ready, they entered their identification code, which was randomly assigned to either the active (response inhibition) or control condition by the computer script using a random number generator. Participants had been told that they would receive either an active or control training task but were given no further information and were therefore blind to condition allocation. After completing the training (10 min), participants were given another set of seven daily FFQs and two 24-h food diaries to complete at home during the intervention (training) week.

Participants were then asked to complete a second, third and fourth training session on their own over the following three days (intervention week^2^). We did not check and encourage compliance (e.g. using reminder phone calls or emails) because we wanted to determine the feasibility of online food no-go training by measuring ‘natural’ rates of compliance. As indicated above, 82% of participants completed all four training sessions with half doing this on the requested days (see supplementary materials). After the intervention week, researchers visited the participants for the third and final time to collect their intervention week FFQs and 24-h food diaries and administer the stimulus evaluation (ratings) test again. Participants then completed the online training task for a final time followed by the taste test. They were also given four filler questionnaires during the taste test (as in Lawrence et al., 2015) to keep them occupied whilst being exposed to the food. Participants were told they could eat as much food as they wanted and were left alone for 15 min, after which the researcher returned, took the food away, weighed the participants and debriefed them. A funnelled debriefing interview (taken from Lawrence et al., 2015) asked participants a series of questions to gauge awareness of the task (stimulus-no-go) associations and to gather feedback about the intervention (see supplementary material).

Finally, participants were asked to complete a short follow-up questionnaire one month and six months after study completion by phone or email, where they provided current (self-reported) weight and monthly FFQ for the past four weeks. Participants were no longer blind to condition allocation at these follow-ups: Due to the probing nature of the debrief interview, participants may have guessed which group they had been allocated to so we decided to un-blind participants during debriefing to standardise awareness. The active participants were given detailed information about the rationale of the training task, however the control participants were informed that general inhibition training may also facilitate weight loss. A small number of participants (16%) voluntarily completed a small number of additional training sessions in-between the one-month and six-month follow-ups. Excluding these participants did not make any difference to the results (see footnotes in Results section).

### 2.10 Power analysis

An a priori power calculation (conducted using G-power 3.1.5) based on data from a single-session food no-go training study (Veling et al., 2011) determined that a total sample of N = 55 would be required to obtain statistical power at the recommended .80 level (Cohen, 1988). Our sample size exceeds this and other single-session lab studies (∼n = 25 per group; Houben & Jansen, 2011; 2015) due to the risk of sample attrition and uncertainty about effect sizes on our real-world dependent variables of weight loss and energy intake.

## 3 Results

All 83 participants with a BMI over 18.5 from whom baseline and post-intervention measures were available were included consistent with an intention-to-treat analysis. Randomization checks showed there were no significant differences between training groups for any potential confounding factors (Table 1).

Our sample reported moderately high scores on disinhibited eating (*M* = 9.12, *SD* = 3.54) compared with previous research in an unselected sample of middle-aged, overweight women from the US (*M* = 6.2, *SD* = 0.2 in Hays et al., 2002). In terms of BMI categories, 22% of participants were a healthy weight (BMI 18.5–24.99), 42% were overweight (BMI 25–29.99) and 36% were obese or morbidly obese (BMI > 30).

### 3.1 Response inhibition (training task) performance

Task performance accuracy in all training sessions was high (at least 80%) demonstrating that all participants were engaged in the training. Supplementary Table 1 displays mean group errors (expressed as a proportion of go and no-go trials) and mean go RT for the first and final training session (completed with the researcher present) to illustrate task performance over time. There were very few errors, performance improved over sessions and there were no differences between groups. Mixed-effects ANOVAs (supplementary materials) confirmed that the active and control groups showed similar task performance and similar improvements over time. Furthermore, both groups showed similar levels of learning of stimulus-specific go- or no-go associations, as demonstrated by the lower error rates and faster reaction times to the 100% go and no-go versus 50% go and no-go-associated stimuli.

### 3.2 Changes in weight

**Fig. 4:**
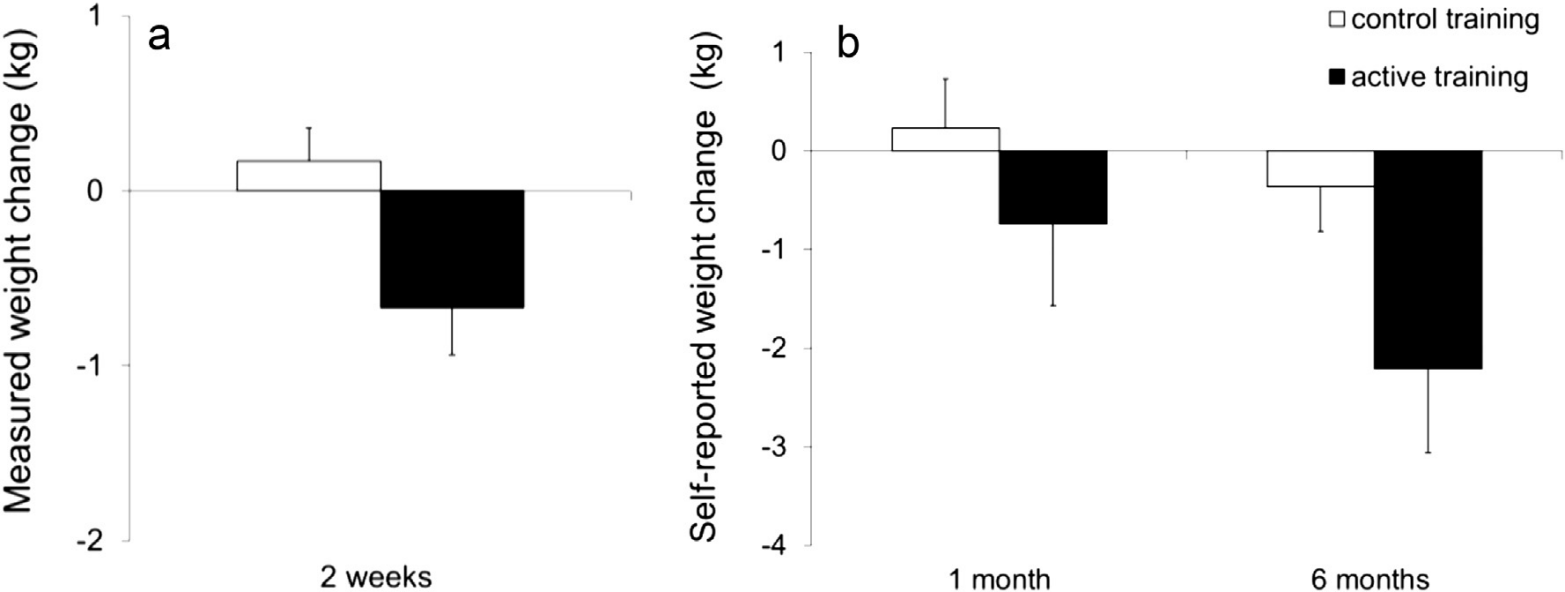
Change in measured weight from baseline to post-intervention (a) and change in self-reported weight from screening to follow-up (b) in each inhibition training condition. A negative change indicates weight loss from pre-to post-intervention. Error bars = standard error of the mean (SEM).

Fig. 4a and b shows changes in measured and self-reported weight at different pre-to post-intervention time-points. The active group showed a reduction in measured weight from baseline to post-intervention (2 weeks), and in self-reported weight from screening to six-month follow-up. Weight changes were analysed in separate 2 × 2 mixed effects ANOVAs comparing baseline to post-intervention, and screening to one-month and six-month follow-up, due to the different weight measures (self-reported instead of researcher-measured weight) for follow-up analyses (Pursey, Burrows, Stanwell, & Collins, 2014). Sample sizes were reduced at follow-up as not all participants were successfully contacted or had weighing scales (see Fig. 1). The two follow-ups were analysed separately due to the inclusion of slightly different participants.

For researcher-measured weight from baseline to post-intervention, there was a significant time × group interaction [F (1, 79) = 6.59, p = .01, η^2^p = .08] but no main effect of time [F (1, 79) = 2.32, p = .13, η^2^p = .03] or group [F (1, 79) = 0.77, p = .38, η^2^p = .01]. As shown in Fig. 4a, the active group lost a significant amount of weight (on average 0.67 kg) over 2 weeks [t (39) = −2.48, p = .02, Cohen's d_z_ = 0.4^3^] whereas weight in the control group increased very slightly (by 0.17 kg) [t (40) = 0.91, p = .37, Cohen's d_z_ = 0.14]. The intervention (between-group) effect for change in weight was of a medium size *d*_s_ = 0.57.

There were no significant changes in self-reported weight from screening to one-month follow-up, shown by non-significant effects of time [F (1,64) = .27, p = .61, η^2^p = .004]; time × group [F (1,64) = 1, p = .32, η^2^p = .015] and group [F(1, 64) = .97, p = .33, η^2^p = .015]. However, there was a reduction in self-reported weight from screening to six-month follow-up,^4^ indicated by an effect of time [F (1,65) = 7.4, p = .008, η^2^p = .1] and a near-significant time × group interaction [F (1,65) = 3.84, p = .054, η^2^p = .056] but no effect of group [F(1, 65) = .96, p = .33, η^2^p = .015]. Fig. 4b shows a significant reduction in self-reported weight in the active group (on average −2.21 kg) over six months [t (31) = −2.6, p = .01, Cohen's d_z_ = 0.47] whereas weight in the control group reduced only slightly (by −0.36 kg) [t (34) = 0.78, p = .44, Cohen's d_z_ = 0.13]. The intervention (between-group) effect on change in self-reported weight at six months was of a medium size *d*_s_ = 0.48. Supplementary table 1 provides details of these and other outcome variables for each group at each time-point.

### 3.3 Changes in snacking frequency

Neither group showed a significant reduction in daily snacking (summed over the four no-go foods) from the baseline week to the intervention week but both groups showed reductions in monthly snacking frequency from screening to the one-month and six-month follow-up (supplementary table 1). Changes in snacking frequency were analysed in separate 2 × 2 ANOVAs from baseline to week 2, screening to one-month, and screening to six-months due to the different (smaller) samples at follow-up, and because monthly rather than daily FFQs were used. Both groups showed small but non-significant reductions in daily snacking from baseline (overall *M* = 6.37, *SD* = 1.27) to week 2 (*M* = 6.21, *SD* = 1.19) [F (1, 80) = 2.34, p = .13, η^2^p = .03]. There was no difference between groups [F (1, 80) = 0.09, p = .77, η^2^p = .001] or time × group interaction [F (1, 80) = 1.18, p = .28, η^2^p = .01]. At one month follow-up there was a significant decrease in monthly snacking relative to screening [F(1,70) = 13.62, p < .001, η^2^p = .16] but no effect of group [F(1,70) = .18, p = .67, η^2^p = .003] or time × group [F(1,70) = 0.07, p = .79, η^2^p = .001]. Similarly at the six-month follow-up^5^ there was a reduction in snacking over the past month relative to screening [F(1,76) = 10.3, p = .002, η^2^p = .12] but no effect of group [F(1,76) = 0.5, p = .48, η^2^p = .006] or time × group [F(1,76) = 0.01, p = .93, η^2^p < .001]. Both groups showed a reduction in monthly FFQ scores from around 15–15.5 at screening to 13.5–14 at each follow-up (supplementary table 1), which is roughly equivalent to reducing intake of three of the no-go snack foods from 2 to 4 times per week at screening to once per week at follow-up.

### 3.4 Changes in energy intake

Daily energy intake (averaged over two 24-h food diaries) was measured during the baseline and intervention week (supplementary table 1). Energy intake showed a reduction in the active group (*M* = −220.4 kcal, *SD* = 514; equivalent to *M* = −922.15 kJ, *SD* = 2150.58) and remained about the same in the control group (*M* = +19.13 kcal, *SD* = 445.12; equivalent to *M* = + 80 kJ, *SD* = 1862.38). This was supported by a time × group interaction [F (1, 78) = 4.96, p = .03, η^2^p = .06], with no effect of group [F (1, 78) = 0.85, p = .36, η^2^p = .01] or reliable effect of time [F (1, 78) = 3.51, p = .065, η^2^p = .04]. Follow-up paired t-tests confirmed a significant drop in energy intake in the active group [t(39) = −2.71, p = .01; Cohen's d_z_ = 0.43] but not in the control group [t(39) = 0.27, p = .79, Cohen's d_z_ = 0.043]. This equated to a medium-sized intervention (between-group) effect on the change in energy intake, d_s_ = 0.5.

### 3.5 Changes in food evaluation

There were a large number of outcome variables in the stimulus evaluation test due to the use of two different ratings (liking and attractiveness), three different categories of food images (healthy-go, high-energy density-no-go and novel foods) and two time points (baseline and post-intervention). To reduce data, we calculated mean change scores from pre-to post-intervention for ratings of liking and attractiveness (separately) for each category of food images. Ratings at baseline were subtracted from ratings post-intervention so that a negative score reflected a drop in ratings over time, consistent with the predicted devaluation effects for no-go foods. Supplementary table 1 provides mean ratings at pre- and post-intervention time-points for each group and stimulus category.

**Fig. 5:**
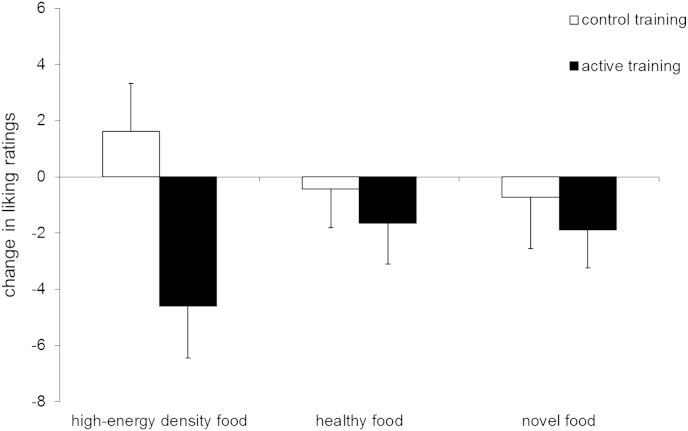
Change in liking ratings from baseline to week 2 as a function of inhibition training condition. Error bars = SEM.

The active group showed a greater reduction in liking than the control group, particularly for high-energy density (no-go) foods (Fig. 5). This was confirmed by a main effect of group [F (1, 78) = 4.13, p = .046, η^2^p = .05] with no effect of food category (3 levels; healthy-go, high-energy density-no-go, novel) [F (2, 77) = 0.06, p = .94, η^2^p = .002] or group × category interaction [F (2, 77) = 1.43, p = .25, η^2^p = .04]. We had specifically predicted a devaluation (reduction in liking) for the high-energy density no-go foods in the active, relative to the control training group (Houben et al., 2012; Veling et al., 2013a), and this was confirmed by a planned between-group t-test [t (78) = −2.49 p = .02, *d*_s_ = 0.56]. As shown in Fig. 5, liking for high-energy density no-go foods decreased in the active training group [paired t (37) = −2.5, p = .02, *d*_z_ = 0.41] and increased slightly (but not reliably) in the control group, [t (41) = 0.96, p = .34, *d*_z_ = 0.15]. There were no differences between groups for changes in liking of healthy or novel foods (ps > .5).

**Fig. 6:**
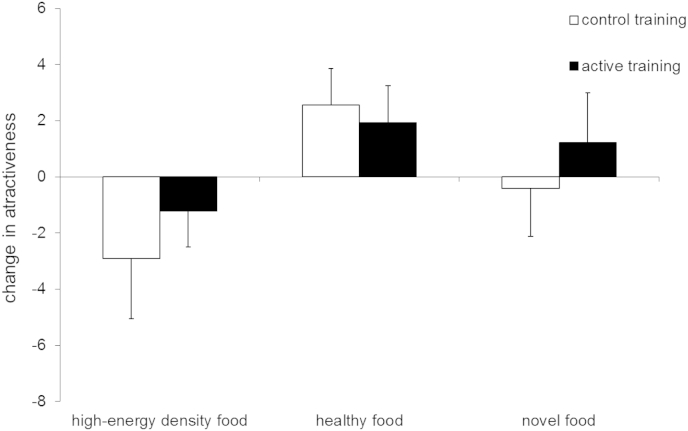
Change in attractiveness ratings from baseline to week 2 as a function of inhibition training condition. Error bars = SEM.

In contrast to the training effects on food liking, both groups showed similar changes in ratings of image attractiveness (Fig. 6), with attractiveness increasing for healthy foods, but decreasing for high-energy density foods. The ANOVA indicated a main effect of stimulus category [F (2, 76) = 4.57, p = .01, η^2^p = .11], but no effect of group [F (1, 77) = 0.3, p = .59, η^2^p = .004] or group × category [F (2, 76) = 0.46, p = .64, η^2^p = .01]. Pairwise contrasts showed that high-energy density and healthy foods (i.e. those presented in the active task) differed significantly for change in attractiveness (p = .003).

### 3.6 Consumption in the taste test

Both groups consumed similar amounts of snack foods (chocolate and crisps) in the taste test after the additional online training session. The active training group consumed a mean total of 187.82 ± 194.71 (SD) kcal, and the control training group consumed a mean of 151.2 ± 122.73 (SD) kcal [t (81) = 1.03, p = .31; Cohen's d_s_ = 0.23].

### 3.7 Task awareness and feedback

During the funnelled debriefing procedure, more than half of active participants (63%) reported noticing that no-go signals or responses were associated with pictures of high-energy density food whereas only 24% of control participants noticed that specific images or categories of objects (e.g. “tools”) were associated with no-go signals or responses. The proportion of “aware” participants was significantly higher in the active than control group (Chi-Square (1, 82) = 12.54, p < .001). We compared active participants who did versus did not report awareness of the associations on our dependent variables of changes in weight, daily calorie intake, high-energy density food liking, and snacking frequency from pre-to post-intervention. There were no significant effects of awareness on any variables (all ps > 0.29) suggesting that explicit awareness did not influence training effects.

Responses in the debriefing interview indicated that more participants in the active group (40%) than in the control group (12%) felt that the task may have influenced their snacking behaviour (Chi-square (1, 82) = 8.5, p = .004) (see supplementary materials for examples of comments). Conversely, a higher proportion of participants (∼50% in each group) reported that the self-monitoring component (daily FFQ and/or food diaries) was “helpful”. Almost all participants said they had no trouble with the training (93%), that they would be prepared to continue doing it if it was effective (88%) and would recommend it to a friend (89%).

### 3.8 Exploratory correlations between outcome measures

We examined whether weight loss at the end of training (and separately at one and six month follow-up) was related to changes in other variables showing intervention effects (changes in snacking frequency, daily calorie intake, liking ratings of high-energy density foods). In the whole sample, self-reported weight loss at one- and six-months was correlated with reductions in daily calorie intake during training (supplementary table 2). These associations were also partly observed within each group (supplementary Tables 3 and 4). There were no other significant correlations between different variables in the whole sample, including no association between changes in daily calorie intake and measured weight loss during training.

Within the active group, there was a moderate but non-significant positive association between the reduction in liking of high-energy density food (devaluation) and measured weight loss at 2 weeks [r(37) = .3, uncorrected p = .075], which was not observed in the control group (supplementary Tables 3 and 4). Changes in food liking did not mediate training effects on measured weight loss (supplementary materials).

### 3.9 Moderation of training effects by BMI

Moderated regression analyses examined whether training effects on measured weight loss were moderated by BMI (as in Veling et al., 2014). The modprobe SPSS macro (Hayes & Matthes, 2009), which explores interactions in multiple regressions, was used with training condition (dummy-coded) as the focal predictor variable, measured weight change at 2 weeks as the dependent variable and baseline BMI as the moderator variable. Results indicated no interaction between training and BMI for weight loss (t (81) = −.36, p = 0.72; Δ R^2^ = 0.002). This suggests that baseline BMI did not influence weight loss during training, which is further supported by non-significant correlations between baseline BMI and weight change in both the active (r (40) = −.03, p = 0.84) and control (r (41) = .05, p = 0.75) groups.

## 4 Discussion

This study examined the feasibility and effectiveness of computerised response inhibition training to food on real-world energy intake and weight loss. Participants completed up to four go/no-go training sessions during the intervention week in either an active (food-associated response inhibition) or control (non-food-associated response inhibition) condition. Participants in the active relative to control condition showed significant weight loss from pre-to post-intervention as well as a reduction in energy intake and liking of high-energy density (no-go) foods. High rates of adherence (97%) and positive feedback suggest the intervention is highly acceptable.

Weight loss from baseline to post-intervention in the active group supports recent research showing that similar food no-go training facilitated weight loss (Veling et al., 2014). Both studies demonstrated similar medium intervention (between-group) effects on weight loss (d_s_ = 0.54 and 0.57) and add to laboratory research showing that food response inhibition training reduces the intake, choice and self-served portion size of no-go foods (Houben, 2011; Houben & Jansen, 2011, 2015; Lawrence et al., 2015; Van Koningsbruggen et al., 2014; Veling et al., 2011, 2013a, 2013b). Self-reported weight loss at six-month but not one-month follow-up in the active group suggests that training effects may persist over longer periods. However, these follow-up data should be interpreted with caution as participants were no longer blind to condition allocation and, whilst self-reported weight is considered a satisfactory measure in web-based weight interventions (Pursey et al., 2014), it can over-estimate intervention effects on weight loss (e.g. Allom & Mullan, 2015). Future studies will therefore need to corroborate these findings using objectively measured weight.

Training effects on measures of eating behaviour were more mixed. Daily snack food intake (FFQs) showed a small but unreliable decrease from the baseline to intervention week and did not differ between groups, supporting similar negative findings from Veling et al. (2014) who used a more comprehensive 24-h FFQ. However, daily calorie intake estimated from 24-h food diaries did show a significant reduction in the active compared to control group, suggesting that food diaries may be a more sensitive and representative measure of daily intake than FFQs (Bingham et al., 1997). FFQs list specific foods only and do not measure portion size (Paalanen et al., 2006); participants may have been consuming smaller portions of snack foods or less of other type(s) of high-energy density food in our study. Interestingly, there were similar significant decreases in monthly snacking frequency at follow-up relative to screening in both groups, suggesting that monthly FFQs may be a more sensitive outcome variable than daily FFQs. This could be due to the summation of subtle changes in snacking frequency over a longer period of time, or because the FFQ at screening was undertaken prior to involvement in the study and therefore participants may have reported higher levels of snacking due to a lack of demand characteristics or self-monitoring (which may have already reduced snacking during the baseline week). The reduction in monthly snacking at follow-up in both groups points to non-specific intervention effects, such as self-monitoring (FFQs and 24-h food diaries), which could have made all participants more aware of their eating behaviour resulting in reduced intake (Burke, Wang, & Sevick, 2011). Consistent with this possibility, during debriefing half of the participants in each group voluntarily reported that they had found the self-monitoring component “helpful”.

The debriefing interviews also revealed that more active (40%) than control (12%) participants thought that the training had influenced their snacking behaviour. This could reflect either subjective awareness of genuine training effects or demand characteristics. We favour the former interpretation as do not believe that many participants knew which group they were in; they had no prior knowledge about this research and were given no information about the different tasks – those in the control group did not know that the active participants were seeing foods in their task, and vice-versa. Participant debriefing from our lab studies suggested that those receiving active training believed that exposure to tasty food pictures in the task may have made them hungrier and eat more food in the subsequent taste test (Lawrence et al. 2015), so one cannot assume that seeing foods made participants aware of which group they were in. Similarly, as many studies have examined the effects of general executive function training (e.g. working memory or response inhibition tasks involving neutral stimuli) on impulsive behaviours (e.g. Houben, Wiers and Jansen, 2011; Bickel, Yi, Landes, Hill, & Baxter, 2011), we felt that the control task was a plausible “brain training” intervention and participants would not necessarily guess they were in the control group.

In terms of possible mechanisms underlying the food no-go training effects, findings from the stimulus evaluation (liking ratings) offer tentative but inconclusive support for stimulus devaluation (Veling et al., 2008, 2013a; Houben et al., 2012). Active training reduced liking of high-energy density no-go foods, and this drop in liking was moderately associated with weight loss in the active group however it did not mediate training effects on weight loss. Perhaps more extensive, sensitive or implicit measures of stimulus evaluation are required to detect mediation effects (Houben et al., 2012). Ratings of image attractiveness also changed pre-to post-intervention (decreasing for high-energy density foods but increasing for healthy foods) however this occurred in both groups, pointing to general intervention effects such as self-monitoring. The different results observed for liking and attractiveness could be linked to, respectively, a greater sensitivity to detect hedonic reactions and motivation to consume the foods (liking of taste) as opposed to general affective responses (attractiveness of image).

The lack of direct correlation between change in daily calorie intake and measured weight loss from pre-to post-intervention also raises questions about the mechanism underlying training effects on weight loss. It has been suggested that 24-h diaries and recalls are a sensitive dietary assessment tool at the group level but not at the individual level, unless multiple recalls are used (Ma et al., 2009). As this was a preliminary study and we wanted to avoid excess burden on participants, we only used two diaries in our pre- and post-intervention week, which may not have provided sufficient sensitivity to detect individual changes that were correlated with weight loss (Pears et al., 2012; Ma et al., 2009). It is also possible that other mechanisms that were not measured, such as changes in exercise, contributed to weight loss.

We did not observe any effects of food response inhibition training on consumption in the taste test in contrast to previous studies (Houben, 2011; Houben & Jansen, 2011, 2015; Veling et al., 2011; Lawrence et al., 2015). Most previous studies were conducted under controlled laboratory conditions where participants were asked not to eat for 2 or 3 h and were seen individually in a lab at specific times of day, whereas the current taste test was conducted in an uncontrolled context (participants' place of work or home) without the time of day, time since last food intake or hunger levels being controlled. These methodological differences may have contributed to lower levels of consumption in the current study (151–187 kcal) compared to the 358–415 kcal consumed in our lab studies that used an identical taste test and very similar response inhibition training (Lawrence et al., 2015). In addition, all lab studies have used control conditions matched for food cue exposure, i.e. control participants had to execute a ‘go’ response to high-energy density foods on at least half of the trials. This may have increased approach motivation towards foods (Schonberg et al., 2014) or primed disinhibition (Guerrieri, Nederkoorn, & Jansen, 2012) and therefore increased the subsequent intake of food in taste tests, confounding the interpretation of results (Lawrence et al., 2015).

BMI did not moderate training effects on weight loss, unlike in a previous study (Veling et al., 2014). However, the current sample was older and more overweight than in Veling et al. (2014) and was similar to their high BMI group. It is possible that once the majority of participants in a sample are overweight (here, 78%), there is no further moderation of food-response inhibition training effects by BMI (i.e. a ceiling effect). Future studies in unselected samples would clarify which factors moderate training effects and for whom such training is likely to be effective.

The current study had a number of limitations. First, it is unclear how participants' self-monitoring interacted with the food response inhibition training – future studies should examine the effects of these factors separately and in combination on weight loss. It would also be useful to measure weight after the baseline week of self-monitoring to measure and control for its effect in both groups. Second, due to time constraints only a limited number of potential mechanisms of training were examined; studies could also examine changes in food-related inhibitory control and motor excitation (Verbruggen & Logan, 2008a; Veling et al., 2011; Chiu et al., 2012, 2014). Future studies could also include additional control conditions matched for food cue exposure (such as passive viewing of the same images presented in the active training task) to control for related processes such as food cue exposure with response prevention. Finally, as food response inhibition training may be especially effective for restrained eaters and chronic dieters (Houben & Jansen, 2011; Veling et al., 2011: Lawrence et al., 2015) studies should continue to examine this in more detail using e.g. the dietary restraint scale (Herman & Polivy, 1980).

Now that promising short-term effects of online food response inhibition training have been established here and in a previous study (Veling et al., 2014) several important issues remain to be examined. First, more objective and detailed measures need to be taken at follow-up to determine longer-term training effects. It would also be useful to examine whether more training sessions conducted over longer periods of time, e.g. 14–25 sessions over 4–6 weeks (Houben et al., 2011; Jones et al., 2014), followed by ‘booster sessions’ at weekly or monthly intervals produces larger and more long-lasting reductions in weight and calorie intake. Future research could also examine the effects of personalized training, whereby participants choose or upload their own high-energy density food images (that they would like to consume less of) as no-go stimuli, and select their own ‘desirable’ low-calorie food images (that they would like to consume more of) as go stimuli. No-go training effects are stronger when foods initially evoke stronger impulses (Veling, Aarts, & Stroebe, 2013b), so personalized training using ‘problem’ foods should be more effective than the standardized training presented here. Another potential research avenue is to examine whether combining food no-go training with other interventions (e.g. implementation intentions as in Veling et al., 2014) or adding more explicit instructions/information about hypothesized mechanisms strengthens training effects. Finally, it would be useful to assess whether alternative methods of delivering the training (e.g. via mobile devices) makes it easier and more accessible, without reducing its effectiveness. In the current sample, 62% of participants thought the training would be acceptable on a smartphone, with some commenting positively on the privacy or convenience of this mode of delivery. The remaining 38% thought smartphone delivery would be problematic, with common reasons including the small size of the screen and buttons, and potential distractions if engaged in other activities or in public. Whilst future empirical research will help to identify how to optimize food no-go training effects, individuals will ultimately choose whether, when and how to do this type of training so offering flexibility may be important.

To conclude, this study suggests that food response inhibition training modifies real-world eating behaviour, reducing calorie intake and facilitating weight loss in a sample of predominantly middle-aged, overweight adults. High rates of adherence and positive feedback suggest the intervention is highly acceptable, and as it could be made freely available online it has the potential to help reduce the burden of overweight and obesity in an accessible and cost-effective manner.

## Figures and Tables

**Table 1:** Participant characteristics per training condition.

	Control (N = 42)	Active (N = 41)	Range	F-value^a^ (p)
Age	51.12 (10.26)	49.79 (9.55)	23–65	0.38 (.54)
Baseline BMI (kg/m^2^)	28.5 (4.71)^b^	29.28 (5.4)^b^	21–46	0.49 (.49)
Sex* (% female)	81	76	N/A	0.35 (.56)
Dieting goal* (% of group)	31	29	N/A	0.03 (.87)
Disinhibition	9.55 (3.71)	8.68 (3.34)	2–16	1.24 (.27)
Monthly snacking	15.62 (3.22)	15.05 (3.15)	9–26	0.67 (.42)
Years education	15.3 (2.3)^c^	15.28 (2.09)^b^	11–19	0.003 (.96)

Note. Standard deviations are presented between parentheses. “Disinhibition” refers to the Three Factor Eating Questionnaire subscale completed at screening, “Monthly snacking” refers to the mean score over the four no-go foods on the FFQ completed at screening.

a Group differences in sex and dieting status (categorical variables) are chi-square values.

b Data missing from one participant in this group.

c Data missing from two participants in this group.

## Footnotes

1 This form is available on the MRC website on dietary assessment; http://dapa-toolkit.mrc.ac.uk/documents/en/EPI/EPIC_24_Hour_Diet_Recall.pdf.

2 We refer to this as the “intervention week” because although participants only completed training on three days, they completed self-monitoring during the whole week and we compared this to the baseline week of self-monitoring.

3 Within-subjects effect-size (d*z*) calculated using method suggested by Lakens (2013).

4 Excluding the 16% of participants who completed extra sessions between one- and six-month follow-ups, the effects were similar; time [F(1, 54) = 8.87, p = .004, η^2^p = .14], time × group [F(1,54) = 5.49, p = .02, η^2^p = .09], group [F(1,54) = 1.15, p = .29, ηp^2^ = .02].

5 Excluding the 16% of participants who did extra training sessions showed similar effects; time [F(1,63) = 6.28, p = .015, η^2^p = .09], group [F(1,63) = .86, p = .36, η^2^p = .01], time × group [F(1, 63) = .08, p = .78, η^2^p = .001].
