# Supplementary material for: Training response inhibition to food is associated with weight loss and reduced energy intake
Source: Appetite. 2015 Dec 1;95:17–28. doi: 10.1016/j.appet.2015.06.009 (PMC4596151; doi:10.1016/j.appet.2015.06.009)
Supplement: Supplementary file 1 [file mmc1.docx]

**Supplementary Material**

**Supplementary Methods**

*Participant recruitment and screening:* Participants were recruited from two community samples of adults through the National Health Service: A local community volunteer research database overseen by the National Institute for Health and Research Exeter Clinical Research Facility (Exeter 10000 - REC: 09/H0106/75, sample 1), and a local mental health Foundation Trust staff mailing list (sample 2). Approximately 8800 individuals were invited by letter or email to complete an online questionnaire about their eating habits, either as part of a study examining the genetics of appetite (c.4500 invited from sample 1; 1203 participants screened) or to assess their suitability for this specific intervention study (c.4300 invited from sample 2; 197 participants screened). The online questionnaire consisted of the Disinhibition subscale from the Three Factor Eating Questionnaire (Stunkard & Messick, 1985), a Food Frequency Questionnaire (FFQ) that assessed the intake of eight high-energy density snack foods over the previous month (Churchill & Jessop, 2011) and three questions related to current dieting, attendance at weight loss groups and use of weight loss pills, along with self-reported height and weight (converted to Body Mass Index, BMI; kg/m2).

Inclusion criteria required that participants snacked at least three times per week (on any of the four ‘no-go’ foods; crisps, chocolate, biscuits and cake) as indicated in the FFQ. One of our dependent variables was the frequency of intake of snack foods presented in the active training task, so we only invited individuals with at least this low level of snacking to participate. In addition, only participants who reported some disinhibition (loss of control over eating) on the Three Factor Eating Questionnaire were invited to participate. This follows evidence that impulsivity and restraint moderate the effects of no-go training in the lab (Houben, 2011; Houben & Jansen, 2011; Lawrence et al., 2015; Veling et al., 2011). As disinhibition is related to impulsivity and scores on the restraint scale (Wardle & Beales, 1987, 1988), as well as to increased BMI and weight gain (Hays et al., 2002), we reasoned that individuals with higher levels of disinhibition might benefit more from food no-go training. Initially, only individuals with a disinhibition score equal to or greater than the sample median (at least 5 out of 16; Mean = 5.5) were invited but due to time constraints, this was reduced to 2 in the later stages of recruitment. However, most of the final sample (89%) scored at least 5 on the disinhibition subscale (see main text Results). Participants also had to be aged 18 – 65 and had to self-report a Body Mass Index (BMI) at screening of at least 18.5 (healthy range and above). We excluded participants with a low BMI due to concerns about potential eating disorders and weight loss in underweight participants. Our sample minimum BMI of 21 suggests eating disorders were not an issue, which was confirmed during debriefing: None of our participants reported any eating disorders, except one control group participant who reported symptoms of bulimia nervosa 38 years previously.

Study exclusion criteria included allergies to the foods given during the taste test (chocolate and crisps) and other factors that could affect weight, namely smoking, recent or present (within the past year) smoking cessation attempts, enrolment in a formal weight-loss programme (e.g. Weightwatchers), use of weight-loss medication, metabolic disorders (e.g. diabetes), allergies to the study foods (chocolate and crisps), and any other health condition that would cause weight-loss.

*Stimulus evaluation test (ratings of food liking and image attractiveness):* For the taste ratings, participants were asked to imagine that some of the food was in their mouth and rate how much they liked the taste. For the attractiveness ratings, participants were asked to rate how attractive the image looked, independent of whether or not they liked the taste of the item. The visual analogue scale was anchored at the extremes with “not at all” and “very much” and participants moved a cursor along the scale using a mouse and pressed the mouse button to confirm their rating. The cursor appeared at the scale mid-point at the beginning of each trial. The evaluation test was administered at baseline and at the beginning of the final session (post-intervention) at the end of week 2. The order of the rating blocks (attractiveness or taste first) was counterbalanced across participants but kept constant within-subject across sessions.

*Food images used in training and ratings tasks:* The food images included in the active training task were as follows: There were 9 high-energy density (greater than 4 kcal/g) no-go food images; crisps (3 exemplars), chocolate (2 exemplars), biscuits (3 exemplars), chocolate cake. There were also 9 healthy go food images; carrot sticks, red pepper, lettuce, grapes, satsuma, apple, rice cakes (2 exemplars), wholemeal cracker. We selected the most frequently consumed snacks based on the FFQ at screening as no-go foods in the active task (these were chocolate, biscuits, cakes and crisps). Some of the food pictures had previously been used in fMRI studies of cue-reactivity, and the food pictures had been rated as pleasant (Beaver et al., 2006; Lawrence et al., 2012). These were supplemented by similar, additional stimuli selected from the internet to ensure sufficient exemplars in each category. The novel food images presented in the stimulus evaluation (ratings) task in addition to the above go and no-go foods included both relatively healthy and high-energy density foods, but these were not firmly separated into healthy vs. high-energy density categories; dried fruit, a sandwich, soup, pizza, jacket potato with butter, mushrooms, quiche, pancakes, vegetarian cannelloni.

*Funnelled debriefing interview:* Researchers asked all participants the following questions during a funnelled debriefing interview:

*Task awareness questions:*

(1) In the computer task did you notice anything in particular? (if “no”, then 2)

(2) For example, did you notice anything about when you had to not press a key? (if “no”, then 3)

(3) Did you think that the stop signals (bold lines) were distributed evenly? (if not, what kind of pictures do you think were associated with the stopping response)?

(4) Do you think that the task influenced your snacking during the week or during today’s session (in the taste test)?

*Feedback on the computerised training*

(5) Did you experience any problems accessing and/or interacting with the training task online?

(6) Were the instructions clear and easy to follow throughout?

(7) Would you be prepared to continue doing this kind of computerised training intervention for a longer period of time?

(8) Do you think this kind of computerised training intervention would be acceptable on a smart phone?

(9) Do you have any feedback / ideas on how we could make the task better/more engaging?

(10) What did you find helpful or unhelpful during participating in this study?

(11) Would you recommend trying this training to friends who wanted to eat fewer snack foods?

*Scoring of participant responses:* Responses from the “awareness” section of the funnelled debriefing interview were coded as follows: Participants who failed to report any awareness of whether some stimuli were more frequently associated with stopping and thought this was completely random were scored “0”, where as those who reported that some specific stimuli (exemplars) were associated with a no-go response or who reported that specific categories of stimuli (e.g. “unhealthy / high-calorie food” or “tools” (in the control task)) were associated with not responding were scored “1”.

*Sample responses (active group) to questions 4 or 10 about whether participants’ felt the training influenced their snacking or was “helpful”:*

“I feel less inclined to reach for biscuits – they are less appealing.” (participant 1)

“The task influenced my snacking – I replaced sweets with strawberries. It is hard to explain why” (participant 6)

“The task made me 'not bothered' about snacking on food – I haven't felt like it. This felt partially conscious but not entirely. I was not eating/seeking snacks.” (participant 16)

“I think it influenced me. Someone gave me chocolate yesterday but I didn't get the same taste I normally would.” (participant 33)

“It made healthy foods more attractive (salad, carrots) than non-healthy. It made me think more about foods I ate.” (participant 38)

“Maybe it affected my chocolate consumption. I had a bar of chocolate at home all week but didn't eat it.” (participant 54)

“It influenced me a couple of times, e.g. didn't eat my cream egg one day (my favourite food) - felt like snacking less. Curious about how it worked.” (participant 56)

**Supplementary Results**

*Compliance with training schedule:* Participants were asked to try and complete additional training sessions on the three consecutive days following their first training session. Of the 83 participants included in the final sample, 40 (48%) followed these instructions precisely and completed three additional sessions on the first, second and third day after their initial training session. An additional 28 participants (35%) completed their training sessions on three other days of the “intervention week” (e.g. the first, third and fourth day), and 15 participants (18%) completed less than three sessions. These patterns of compliance were similar in the active and control groups. Four of the 83 participants completed two sessions in one day (2 participants in each group).

*Statistical analysis of response inhibition training performance over time:* Supplementary Table 1 (below) displays mean task performance for each group in the first and final training session. Mixed-effects ANOVA confirmed that go errors improved over time [F (1, 81) = 25.16, p < .001, η^2^p = 0.24] but showed no difference between groups [F (1, 81) = 2.14, p = .15, η^2^p = .03], or group x time interaction [F (1, 81) = 0.25, p = .62, η^2^p = .003]. For no-go errors, we included a second within-subjects factor of stimulus category in the ANOVA with two levels; high-energy density foods (or their control training equivalents) and non-food. This enabled a comparison of no-go errors to stimuli that were 100% associated with no-go signals (high-energy density foods or their control equivalents) relative to stimuli that that were 50% associated with no-go signals (the non-food filler pictures of clothing). No-go error rates improved over time [F (1, 81) = 95.07, p < .001, η^2^p = .54] but did not differ as a function of group [F (1, 81) = 0.58, p = .45, η^2^p = .007] or group x time [F (1, 81) = 0.44, p = .51, η^2^p = .005]. There was a main effect of stimulus category [F (1, 81) = 23.41, p < .001, η^2^p = .22], with fewer no-go errors to the 100% no-go stimuli (high-energy density food or their control equivalents) than to the 50% no-go non-food stimuli (see supplementary table 1) but category did not interact with group [F (1, 81) = 0.59, p = .45, η^2^p = .007], time [F (1, 81) = 2.84, p = .1, ηp2 = .034] or group x time [F (1, 81) =0.85, p = .36, η^2^p = .01].

Go RT became significantly faster over time [F (1, 81) = 113.75, p < .001, η^2^p = .58] but did not differ as a function of group [F (1, 81) = 0.26, p = .61, η^2^p = .003] or group x time [F (1, 81) = 2.32, p = .13, η^2^p = .028]. There was a main effect of stimulus category on Go RT [F (1, 81) = 89.18, p < .001, η^2^p = .52], with faster RTs to the 100% go stimuli (healthy food or their control equivalents) than to the 50% go non-food filler stimuli (see supplementary table 1). There was also a stimulus category x time interaction [F (1, 81) = 15.43, p < .001, η^2^p = .16] due to a larger speeding-up of go RTs to the non-food clothing stimuli, perhaps due to faster RTs (floor effects) for the food stimuli.

*Mediation analysis:* We tested whether the effect of active vs. control training on weight loss was mediated by the devaluation (drop in liking) of no-go foods. Weight and food liking data were standardized, the change scores (from baseline to week 2) were computed and the indirect SPSS macro (Preacher & Hayes, 2008) was used to estimate direct and indirect effects of training on weight loss (with 1000 bootstrap samples). Training condition (dummy-coded, with active training coded “1”) was the independent variable, change in weight (2 weeks minus baseline) was the dependent variable and change in liking (2 weeks minus baseline) was the mediator variable. As can be seen in supplementary figure 1, active training was related to a reduction in weight and to a reduction in food liking, but the change in liking did not mediate the direct influence of training on weight loss. The 95% confidence interval of the bootstrapping analysis for the indirect effect included zero (range: -.03-.003).


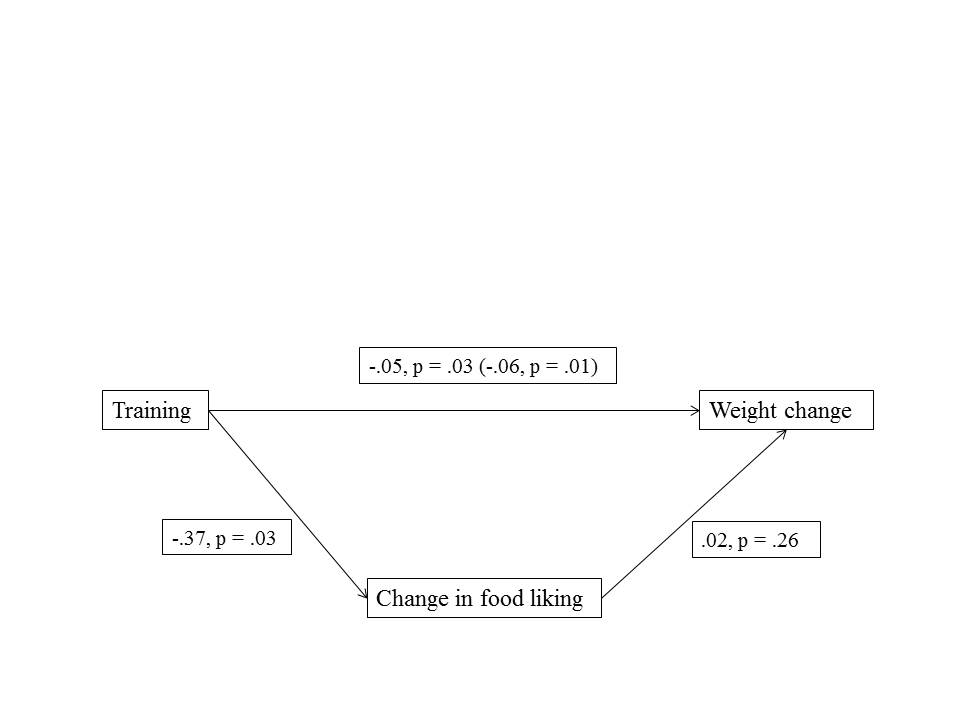


**Supplementary Figure 1 Result of the mediation analysis.** Note: path values reflect standardized regression coefficients. The value in parentheses represents the total effect of training on weight loss of the bootstrapping analyses. Values outside parentheses reflect the direct effects of the bootstrapping analyses.

**Supplementary References (not in main manuscript)**

Beaver, J., Lawrence, A., van Ditzhuijzen, J., Davis, M., Woods, A., & Calder, A. (2006). Individual Differences in Reward Drive Predict Neural Responses to Images of Food. *The Journal of Neuroscience*, 26, 5160 - 5166.

Preacher K. J., & Hayes A. F. (2008). Asymptotic and resampling strategies for assessing and comparing indirect effects in multiple mediator models. *Behavior Research Methods*, 40, 879–891.

Wardle, J., & Beales, S. (1987). Restraint and food intake: An experimental study of eating patterns in the laboratory and in normal life. *Behaviour Research and Therapy*, 25, 179 - 185.

Wardle, J., & Beales, S. (1988). Control and loss of control over eating: An experimental investigation. *Journal of Abnormal Psychology*, 1, 35 - 40.

**Supplementary Table 1**

*Descriptive information, significance tests between conditions, and number of participants*

Time-point Baseline Post-training Baseline Post-training

Group Active Active Control Control *F-*tests (n=41) (n=41) (n=42) (n=42) (Group effect)

Go errors (All) .033 (.043) .009 (.022) .024 (.032) .005 (.007) F=2.14, p=.15

No-Go errors (UF) .025 (.023) .012 (.017) .025 (.02) .004 (.009) F=1.85, p=.18

No-Go errors (NF) .041 (.03) .014 (.018) .039 (.033) .015 (.019) F=0.01, p=.92

Go Reaction time ms (HF) 587.45 (93.79) 523.46 (105.9) 606.91 (86.27) 520.86 (81.85) F=0.21, p=.65

Go Reaction time ms (NF) 615.11 (92.06) 537.81 (111.15) 638.37 (81.65) 536.08 (88.08) F=0.32, p=.58

Weight kg 83.91 (14.9)^1^ 83.24 (14.88)^1^ 80.54 (15.28)^1^ 80.71 (15.36)^1^ *F*=1.1, *p*=.34

Weight kg (1 month)* 83.46 (16.99)^8^ 82.72 (15.21)^8^ 79.06 (16.12)^9^ 79.3 (16.6)^9^ *F*=0.97, *p*=.33

Weight kg (6 months)* 83 (17.27)^9^ 80.79 (14.86)^9^ 78.31 (15.51)^7^ 77.96 (15.49)^7^ *F*=0.96, *p*=.33

Daily FFQ 6.28 (1.23) 6.23 (1.21) 6.47 (1.32)^1^ 6.18 (1.19)^1^ F=.085, p=.77

Monthly FFQ (1 month) 15.03 (3.26)^3^ 13.55 (3.9)^3^ 15.44 (3.33)^8^ 13.74 (3.36)^8^ F=.18, p=.67

Monthly FFQ (6 months) 15.05 (3.2)^2^ 13.62 (3.88)^2^ 15.56 (3.08)^3^ 14.05 (4.15)^3^ F=.5, p=.48

Energy intake kcal (24-hour) 2233.58 (551.07)^1^ 2013.16 (570.98)^1^ 2006.74 (515.14)^2^ 2025.87 (644.48)^2^ F=.85, p=.36

(kJ 9345.3 (2305.68) ^1^ 8423.06 (2389) ^1^ 8396.2 (2155.3) ^2^ 8476.24 (2696.5) ^2^)

Liking (UF-no-go) 67.2 (14.43)^3^ 62.61 (14.49)^3^ 62.13 (14.99) 63.76 (14.72) *F*=6.2, *p*=.015

Liking (HF-go) 56.31 (14.32)^3^ 54.67 (12.54)^3^ 55.8 (13.98) 55.37 (14.84) *F*=.37, *p*=.55

Liking (novel food) 67.38 (9.83)^3^ 65.49 (11.01)^3^ 67 (12.27) 66.28 (13.42) *F*=.4, *p*=.53

Attractive (UF-no-go) 50.06 (14.19)^4^ 48.84 (14.91^)4^ 48.14 (12.35) 45.24 (15.66) *F*=.42, *p*=.52

Attractive (HF-go) 53.66 (12.05)^4^ 55.59 (11.79)^4^ 50.34 (11.13) 52.9 (9.46) *F*=.11, *p*=.74

Attractive (novel food) 51.19 (11.2)^4^ 52.42 (10.7)^4^ 48.74 (11.53) 48.33 (10.65) *F*=.44, *p*=.51

Food intake kcal (taste test) 187.82 (194.7) 151.21 (122.73) *F*=1.06, *p*=.31

Task awareness (% of sample) 63 24 χ^2^12.5,p<.001

Note: Differences reported in text were tested in mixed-effects ANOVAs for time (baseline vs. post-training) and group. Standard deviations are given between parentheses. Errors = proportion of trials with incorrect response. UF = Unhealthy foods or their control task equivalents; NF = non-food clothing (filler) stimuli; HF = healthy food images or their control task equivalents. FFQ = Food frequency questionnaire. * = Weight for 1 month and 6 month follow-ups was self-reported and “baseline” weight for these data is self-reported weight at screening, ^1^Data missing from one participant in this cell, ^2^Data missing from 2 participants. ^3^Data missing from 3 participants, etc. All remaining superscript numbers indicate the number of missing data points for this cell (these range from 2-9 for 1- and 6-month follow-up data).

**Supplementary Table 2: Correlations between outcome measures in whole sample**

|  | | Weight change (training week) | | Weight change (one month) | | Weight change (six months) | Daily snacking change (training week) | Monthly snacking change (one month) | Monthly snacking change (six months) | Change in daily energy intake (training week) |
| --- | --- | --- | --- | --- | --- | --- | --- | --- | --- | --- |
| Weight change (one month follow-up) | Pearson r | | .11 | |  |  |  |  |  |  |
|  | p-value | | .37 | |  |  |  |  |  |  |
|  | N | | 65 | |  |  |  |  |  |  |
| Weight change (six months follow-up) | Pearson r | | .16 | | **.74^**^** |  |  |  |  |  |
|  | p-value | | .19 | | **<.001** |  |  |  |  |  |
|  | N | | 66 | | 59 |  |  |  |  |  |
| Daily snacking change (training week) | Pearson r | | .05 | | .11 | .17 |  |  |  |  |
|  | p-value | | .65 | | .38 | .17 |  |  |  |  |
|  | N | | 80 | | 65 | 66 |  |  |  |  |
| Monthly snacking change (one month follow-up) | Pearson r | | .23 | | -.06 | .07 | .04 |  |  |  |
|  | p-value | | .05 | | .63 | .61 | .73 |  |  |  |
|  | N | | 69 | | 60 | 56 | 70 |  |  |  |
| Monthly snacking change (six months follow-up) | Pearson r | | .03 | | -.10 | .06 | .16 | **.53^**^** |  |  |
|  | p-value | | .80 | | .44 | .61 | .17 | **<.001** |  |  |
|  | N | | 76 | | 62 | 67 | 77 | 67 |  |  |
| Change in daily energy intake (training week) | Pearson r | | .15 | | **.36**** | **.35**** | .10 | .05 | .12 |  |
|  | p-value | | .18 | | **.004** | **.005** | .40 | .70 | .32 |  |
|  | N | | 80 | | 64 | 65 | 79 | 68 | 75 |  |
| Liking change (high-energy density foods) | Pearson r | | .19 | | .06 | .15 | -.01 | -.07 | -.04 | *.215* |
|  | p-value | | .10 | | .66 | .24 | .94 | .58 | .76 | *.06* |
|  | N | | 78 | | 63 | 64 | 79 | 68 | 75 | 77 |

Note: Trend-level correlations with changes in liking in italics. * p < .05, ** p < .001

**Supplementary Table 3:** **Correlations between outcome measures in active group**

|  | | Weight change (training week) | Weight change (one month) | Weight change (six months) | Daily snacking change (training week) | Monthly snacking change (one month) | Monthly snacking change (six months) | Change in daily energy intake (training week) |
| --- | --- | --- | --- | --- | --- | --- | --- | --- |
| Weight change (one month follow-up) | Pearson r | .15 |  |  |  |  |  |  |
|  | p-value | .41 |  |  |  |  |  |  |
|  | N | 33 |  |  |  |  |  |  |
| Weight change (six months follow-up) | Pearson r | .07 | **.86**** |  |  |  |  |  |
|  | p-value | .69 | **<.001** |  |  |  |  |  |
|  | N | 32 | 29 |  |  |  |  |  |
| Daily snacking change (training week) | Pearson r | .06 | .19 | **.36^*^** |  |  |  |  |
|  | p-value | .73 | .30 | **.04** |  |  |  |  |
|  | N | 40 | 33 | 32 |  |  |  |  |
| Monthly snacking change (one month follow-up) | Pearson r | .22 | -.12 | -.04 | .18 |  |  |  |
|  | p-value | .2 | .52 | .84 | .28 |  |  |  |
|  | N | 36 | 31 | 28 | 37 |  |  |  |
| Monthly snacking change (six months follow-up) | Pearson r | .11 | -.18 | .07 | .27 | **.54^**^** |  |  |
|  | p-value | .50 | .33 | .72 | .10 | **.001** |  |  |
|  | N | 38 | 31 | 32 | 39 | 35 |  |  |
| Change in daily energy intake (training week) | Pearson r | .15 | .31 | **.37*** | .06 | .11 | .19 |  |
|  | p-value | .37 | .075 | **.04** | .70 | .53 | .24 |  |
|  | N | 40 | 33 | 32 | 40 | 36 | 38 |  |
| Liking change (high-energy density foods) | Pearson r | *.30* | -.13 | -.04 | .22 | .03 | .03 | .16 |
|  | p-value | *.075* | .50 | .83 | .18 | .87 | .88 | .35 |
|  | N | *37* | 30 | 29 | 38 | 34 | 36 | 37 |

Note: Trend-level correlations with changes in liking in italics. * p < .05, ** p < .001

**Supplementary Table 4: Correlations between outcome measures in control group**

|  | | Weight change (training week) | Weight change (one month) | Weight change (six months) | Daily snacking change (training week) | Monthly snacking change (one month) | Monthly snacking change (six months) | Change in daily energy intake (training week) |
| --- | --- | --- | --- | --- | --- | --- | --- | --- |
| Weight change (one month follow-up) | Pearson r | -.12 |  |  |  |  |  |  |
|  | p-value | .53 |  |  |  |  |  |  |
|  | N | 33 |  |  |  |  |  |  |
| Weight change (six months follow-up) | Pearson r | .08 | **.40*** |  |  |  |  |  |
|  | p-value | .66 | **.03** |  |  |  |  |  |
|  | N | 34 | 30 |  |  |  |  |  |
| Daily snacking change (training week) | Pearson r | .13 | .05 | .02 |  |  |  |  |
|  | p-value | .41 | .81 | .90 |  |  |  |  |
|  | N | 40 | 32 | 34 |  |  |  |  |
| Monthly snacking change (one month follow-up) | Pearson r | .32 | .06 | .34 | -.12 |  |  |  |
|  | p-value | .07 | .77 | .08 | .50 |  |  |  |
|  | N | 33 | 29 | 28 | 33 |  |  |  |
| Monthly snacking change (six months follow-up) | Pearson r | -.13 | .001 | .04 | .05 | **.54^**^** |  |  |
|  | p-value | .45 | .99 | .82 | .78 | **.002** |  |  |
|  | N | 38 | 31 | 35 | 38 | 32 |  |  |
| Change in daily energy intake (training week) | Pearson r | -.01 | **.38*** | .14 | .20 | -.04 | .03 |  |
|  | p-value | .98 | **.03** | .44 | .22 | .82 | .85 |  |
|  | N | 40 | 31 | 33 | 39 | 32 | 37 |  |
| Liking change (high-energy density foods) | Pearson r | -.08 | .29 | .32 | -.20 | -.15 | -.11 | .16 |
|  | p-value | .62 | .10 | .06 | .22 | .41 | .50 | .31 |
|  | N | 41 | 33 | 35 | 41 | 34 | 39 | 40 |

Note * p < .05, ** p < .001
